# Association Between Organizational Bullying, Moral Distress, and Disengagement Among Emergency Department Nurses in Ilam Hospitals: A Cross Sectional Study

**DOI:** 10.1155/jonm/5230876

**Published:** 2025-10-31

**Authors:** Laya Besharaty, Alun C. Jackson, Zahra Rahmaty, Mohammad Namazi Nia, Fatemeh Bahramnezhad

**Affiliations:** ^1^Department of Emergency Nursing, Nursing and Midwifery Care Research Center, School of Nursing & Midwifery, Tehran University of Medical Sciences, Tehran, Iran; ^2^Centre on Behavioral Health, University of Hong Kong, Pokfulam, Hong Kong, China; ^3^Faculty of Human and Health Sciences, School of Nursing, University of Northern British Columbia, Prince George, Canada; ^4^Department of Nursing, School of Nursing and Midwifery, Nohom-e-Dey, Torbat Heydariyeh University of Medical Sciences, Mashhad, Iran; ^5^Department of Critical Care Nursing, Nursing and Midwifery Care Research Center, School of Nursing & Midwifery, Tehran University of Medical Sciences, Tehran, Iran

**Keywords:** emergency nurse, Ilam, Iran, moral disengagement, moral distress, organizational bullying

## Abstract

**Background:**

It is widely accepted that emergency department is an inherently high-stress environment where organizational bullying is prevalent, potentially leading to psychological harm and ethical complications. Moral distress occurs when nurses feel unable to adequately resolve ethical challenges, while moral disengagement involves mental processes that rationalize or excuse unethical actions, thereby diminishing personal responsibility.

**Methods:**

This descriptive-analytical study was carried out in 2024 across public and private hospitals in Ilam Province, a border region in western Iran. Ilam Province has a total of 11 hospitals, consisting of 9 public hospitals and 2 private hospitals. The study population comprised 266 nurses working in emergency departments, with the entire group selected via census sampling. The research instruments included the Einarsen Organizational Bullying Questionnaire, the Hamric Moral Distress Scale, and Fida's Moral Disengagement in Nursing instrument. Data were analyzed using SPSS version 25, examining the complex relationships among organizational bullying, moral distress, and moral disengagement. Model 4 of the PROCESS macro was employed to assess mediation effects. Descriptive statistics were calculated for all variables, and data normality was assessed using skewness and kurtosis values. To further explore the associations among the key variables, Pearson correlation coefficients were computed.

**Results:**

The nurses' mean ratings for organizational bullying, moral distress, and moral disengagement were 42 ± 14.5, 22.2 ± 12.8, and 39 ± 10.9, respectively. Statistical research found a moderate link between organizational bullying and moral suffering (p = 0.0112, *r* = 0.1865). Furthermore, a substantial and direct relationship was detected between moral discomfort and moral disengagement (*p* < 0.001, *r* = 0.6470). A positive and moderate connection was discovered between organizational bullying and moral disengagement (*p*=0.0006, *r* = 0.2358).

**Conclusion:**

The findings of this study show that organizational bullying is strongly linked to moral anguish among emergency department nurses. Furthermore, moral distress has a significant and direct association with moral disengagement, suggesting that it may weaken ethical awareness and increase the likelihood of unethical behavior in the workplace. Furthermore, the considerable positive link between organizational bullying and moral disengagement suggests that increasing bullying behaviors may exacerbate moral disengagement among nurses. These findings highlight the necessity of adopting organizational measures to minimize bullying in healthcare settings while also improving nurses' psychological and ethical well-being. It is recommended that hospital administrators and health system policymakers develop and implement educational and supportive programs aimed at mitigating organizational bullying, bolstering moral resilience, and promoting the overall professional well-being of nurses.

**Implications for Nursing Management:**

The findings indicate that organizational bullying significantly influences moral distress and moral disengagement among nurses, highlighting the need for targeted management strategies. Nursing leaders should implement strict antibullying policies, foster ethical decision-making through training programs, and provide psychological support systems to reduce moral distress. Additionally, mentoring younger and less experienced nurses, particularly those in public hospitals and emergency departments, can help mitigate disengagement. Creating a culture of accountability, promoting ethical leadership, and ensuring open communication will enhance job satisfaction and improve patient care outcomes.

## 1. Introduction

As the largest professional group in the healthcare system, nurses are frequently exposed to various psychological and physical challenges in their work environment [1–3]. These difficulties are especially intensified in emergency departments, which handle approximately 78% of all hospital admissions [3]. Factors such as excessive workload, extended shifts, and inadequate staffing contribute not only to burnout and mental exhaustion but also to decreased quality of patient care [4, 5], while simultaneously elevating the risk of workplace violence and organizational bullying [6].

Organizational bullying—identified by the World Health Organization (WHO) as a significant public health issue [7]—refers to the repeated and systematic expression of interpersonal aggression within the workplace. This phenomenon inflicts both psychological and physical harm, diminishes job satisfaction, and exacerbates occupational stress [8–12]. This phenomenon has been referred to as a “silent global epidemic.” An unhealthy work environment and organizational bullying can be considered one of the greatest threats to employee well-being and may exacerbate workforce shortages, particularly in the nursing profession [2, 5].

Various studies have reported a wide range in the prevalence of organizational bullying, from 3.9% to 86.5% [8]. However, what is most significant is that organizational bullying has evolved into a persistent and systematic form of interpersonal violence, in which the victim is subjected to prolonged negative behaviors—most often from a superior—and is unable to defend themselves [8].

Bullying frequently manifests through repeated verbal abuse, social exclusion, unreasonable and threatening task assignments, mockery, intimidation, and humiliation by supervisors [9, 10].

Several causes have been cited for this phenomenon, including excessive workload, aggression from patients or their companions, the presence of multiple ethical challenges, a negative organizational climate, and authoritarian or discriminatory managers—all of which impair interpersonal relationships and lead to bullying from peers or superiors [13, 14].

Organizational bullying is destructive and can result in physical and psychological harm to the victim, reduced productivity, and increased turnover and job instability. It also contributes to heightened stress, decreased job satisfaction, reduced organizational commitment and performance, and a diminished quality of life for employees [10, 15].

In severe cases, victims of bullying may even attempt suicide [11]. A direct link has been established between bullying in the workplace and increased rates of turnover and intention to leave [11, 16, 17].

Importantly, the victim is not the only one affected. Over time, the perpetrator may also experience adverse consequences such as anxiety, depression, sleep disturbances, irritability, difficulty concentrating, somatic complaints (e.g., bodily pain and fatigue), and gastrointestinal symptoms [9].

Organizational bullying also has broader negative impacts, such as increased workload pressure, reduced work motivation, diminished concentration, physical fatigue, sleep difficulties, and a variety of mental health disorders including depression, anxiety, post-traumatic stress disorder (PTSD), suicidal ideation, and impaired immune function. These consequences, in turn, reduce patient safety and the quality of healthcare services provided by nurses [11, 18, 19].

Many scholars argue that these effects are not limited to victims alone. Bullies themselves may also experience long-term consequences such as lower self-esteem, anxiety, exhaustion, negative emotions, social withdrawal, and dishonesty. These outcomes have a negative impact on the organization as a whole, reducing job satisfaction, motivation, performance, organizational commitment, and sense of belonging [11, 12].

According to previous studies, organizational bullying occurs more frequently in healthcare settings than in other workplaces. This is thought to be due to more intense interpersonal interactions, the emotional demands of caregiving, hierarchical organizational structures, and conflicting priorities within healthcare teams [8, 11, 20].

The main sources of bullying against nurses have been identified as managers and supervisors [12], as well as more experienced nurse colleagues [20].

Furthermore, the occurrence of bullying is influenced by cultural factors and the criteria used for managerial selection. When an unqualified individual is placed in a managerial role, they may exhibit bullying behaviors toward subordinates due to awareness of their own shortcomings [21].

Therefore, effective interventions to prevent bullying must be grounded in a contextual understanding of each organization. Conducting organization-specific studies to identify the underlying causes of bullying is essential [20].

Beyond the aforementioned physical and psychological consequences, organizational bullying can also lead to ethical challenges, particularly moral distress among nurses [22].

For nurses, such stressful environments may result in moral distress, a concept defined by Hamric as the psychological discomfort experienced when individuals recognize the ethically appropriate action to take but are constrained from acting due to institutional or hierarchical barriers [13, 14]. This inner conflict leads to feelings of frustration, sadness, and powerlessness and may ultimately reduce the quality of care provided and prompt some nurses to leave the profession altogether [15–17]. From a professional perspective, moral distress can lead to reduced nurse–patient communication, as the nurse may avoid contact with the patient and lose the ability to provide safe care. Ultimately, this becomes an additional contributing factor to job turnover [23, 24]. These outcomes are similar to those experienced by individuals subjected to workplace bullying. When moral distress and bullying occur simultaneously—or when bullying triggers moral distress—the severity of the process is amplified [25].

In contrast to moral distress stands moral disengagement, which is considered a social-cognitive strategy. It refers to one or more cognitive mechanisms used to selectively deactivate moral self-regulatory processes that would normally inhibit unethical behavior [22].

Some individuals may resort to moral disengagement—a psychosocial mechanism described by Bandura, which enables people to distance themselves from their internal moral standards in order to avoid feelings of guilt [26]. Beyond its psychological consequences, moral disengagement also influences the spiritual, emotional, and social dimensions of well-being [27]. Under specific circumstances, individuals may engage in behaviors that conflict with their ethical beliefs while remaining emotionally detached from the moral implications of their actions [28, 29]. Moral disengagement can act as a significant factor in reducing positive ethical behaviors such as empathy and can increase the likelihood of behaviors like cynicism [30], antisocial behaviors [31], aggression [32], and Machiavellianism (disregard for ethical norms to achieve personal goals) [31]. Additionally, Detert et al. stated that moral disengagement can increase the probability of unethical decision-making [33]. On the other hand, a study by Fida et al. showed that stressful work environments and the negative emotions experienced by individuals working in these settings can increase the likelihood of moral disengagement [34].

Given these concerns, a key question emerges: How are moral distress and moral disengagement among nurses associated with organizational bullying in Ilam? Current literature offers limited insights into the interplay of these factors, underscoring the need for further investigation. During their time as both a student and practicing nurse, the researcher has directly observed a growing trend of nurse attrition, particularly in emergency departments. Ilam Province, located in western Iran and home to over 550,000 residents across 12 counties, is one of the most underserved regions in the country despite its rich oil and gas reserves. Official reports indicate high rates of poverty and unemployment, while suicide ranks as the third leading cause of death among individuals aged 15–24—placing the province among those with the highest suicide rates both nationally and, in some years, globally [35, 36]. Despite extensive research on organizational bullying and moral distress individually, there remains a significant gap in understanding how these phenomena interact with moral disengagement, particularly within the unique and high-pressure context of emergency department nurses. This gap is especially critical in underserved regions like Ilam Province, where healthcare challenges are compounded by socioeconomic hardships and elevated mental health risks, including high suicide rates. Exploring the interrelations among organizational bullying, moral dilemma, and moral disengagement in this setting is essential to develop targeted interventions that not only improve nurse well-being and retention but also enhance the quality of patient care and safety in environments facing resource limitations and cultural complexities. Thus, this study aims to fill an important gap by providing empirical evidence on these interconnected constructs in a context that has been largely overlooked in the literature.

### 1.1. Purpose

This study, conducted in 2024, aimed to determine the relationship between organizational bullying and both moral distress and moral disengagement among nurses working in the emergency departments of hospitals in Ilam Province.

## 2. Methods

### 2.1. Study Design and Participants

This cross-sectional study was conducted in 2024 to determine the relationship between organizational bullying and both moral distress and moral disengagement among nurses working in the emergency departments of hospitals in Ilam Province. Ilam Province has a total of 11 hospitals, consisting of 9 public hospitals and 2 private hospitals. The study population consisted of nurses employed in the emergency departments of both public and private hospitals in Ilam.

### 2.2. Sample Size

A sample size of 277 participants was calculated. Given that 320 nurses were working in the emergency departments and considering that some might not meet the inclusion criteria or might be unwilling to participate, all emergency department nurses were included in the study through census sampling. Pregnant nurses and those on any form of extended leave (such as maternity, medical, or personal leave) at the time of data collection were excluded from the study. This decision was made because their temporary absence from the clinical environment could influence their experience of moral distress.

### 2.3. Data Collection Instruments

#### 2.3.1. Demographic Questionnaire

This questionnaire collected data on age, gender, marital status, level of education, shift rotation, years of nursing experience, length of service in the emergency department, and hospital type.

#### 2.3.2. Organizational Bullying Questionnaire

Developed by Einarsen et al. in 2009, this questionnaire consists of 22 items and is structured into three components: work-related bullying, personal bullying, and physical bullying. Responses are recorded on a 5-point Likert scale, yielding a total score ranging from 22 to 110. A higher score indicates a greater level of organizational bullying [37, 38]. In the present study, the content validity of the tool was confirmed, and its reliability was assessed using Cronbach's alpha, which was calculated at 0.89.

#### 2.3.3. Revised Moral Distress Questionnaire

Originally designed by Hamric et al. in 2012, this questionnaire initially contained 21 items. In Iran, Arab and Barzegar translated and validated the instrument in 2014; subsequently, three items were removed, resulting in an 18-item tool. Each item is rated on a Likert scale ranging from 0 to 4, and two dimensions—level of disturbance and frequency—are measured for every item. The overall score ranges from 0 to 288, with scores categorized as low (0–96), moderate (97–192), and high (193–288), where a higher score indicates a more severe moral distress. In the study conducted by Arab and Barzegar, the reliability of the instrument was reported as Cronbach's alpha of 0.75‴ [26].

#### 2.3.4. Nursing Moral Disengagement Questionnaire

Developed by Fida et al. in 2015, this questionnaire consists of 22 items and is unidimensional, with responses captured on a 5-point Likert scale. The total score ranges from 22 to 110, with higher scores reflecting greater moral disengagement among nurses [39]. Since this instrument had not previously been psychometrically evaluated in Iran, it was translated using a backward–forward translation approach. For content validity, the questionnaire was reviewed by ten faculty members from the Nursing and Midwifery School of Tehran and their suggestions were incorporated outside. To assess reliability, the instrument was administered to 20 individuals who were not part of the study sample, and Cronbach's alpha of 0.85 was subsequently calculated.

### 2.4. Data Collection

After obtaining approval for the research proposal and ethical clearance from the Tehran University of Medical Sciences (IR.TUMS.FNM.REC.1403.012), the questionnaires were distributed in paper format through census sampling across the hospitals, following confirmation of their content validity. The researcher, after obtaining the necessary permissions and coordinating accordingly, regularly attended various shifts in the emergency departments. Upon securing written informed consent, the paper questionnaires were provided to eligible nurses.

Inclusion criteria consisted of having a minimum of 6 months' experience in the emergency department and willingness to participate. Exclusion criteria involved failure to complete at least 70% of the questionnaire items, while nonentry criteria included a history of severe stress, substance abuse (drugs and alcohol), and the use of antianxiety and antidepressant medications.

### 2.5. Data Analysis

The data were analyzed using SPSS Statistics (version 16.0) and the PROCESS macro (version 4.2) by Hayes. To examine the complex relationships among organizational bullying, moral distress, and moral disengagement, Model 4 of the PROCESS macro was employed to assess mediation effects. This model allowed us to evaluate both the direct and indirect pathways, as well as the total effects of organizational bullying on moral disengagement, with moral distress acting as a potential mediator.

Descriptive statistics were calculated for all variables, and data normality was assessed using skewness and kurtosis values. The main variables were found to be normally distributed, validating the assumptions required for mediation analysis.

To further explore the associations among the key variables, Pearson correlation coefficients were computed, revealing significant relationships between organizational bullying, moral distress, and moral disengagement. Mediation analysis was then conducted using bias-corrected bootstrapping with 5000 resamples to estimate the indirect effects. A 95% confidence interval (CI) was used, and indirect effects were deemed statistically significant when the CI did not include zero.

All analyses were adjusted for relevant covariates, including age, gender, education level, marital status,…, to account for potential confounding variables. The results provide robust insights into the mediating role of moral distress in the relationship between organizational bullying and moral disengagement.

## 3. Results

Out of the total of 266 nurses who participated in the study, the highest frequency was observed among 152 nurses (57.1% male) and 147 nurses (55.2%) aged 23–30; the remaining demographic characteristics are detailed in Table 1.

An evaluation of the nurses' scores revealed that the mean organizational bullying score was 42 ± 14.5, with the highest subscale average recorded in the personal bullying dimension (22.3 ± 6.3). The findings further indicated that there was no significant relationship between organizational bullying and the demographic variables assessed in the study.

The mean moral distress score among the nurses was 22.2 ± 12.8. Breaking this down, the average for the disturbance component was 22.3, and for the frequency component, it was 22.1. Most nurses fell within the low moral distress category. Moreover, results showed significant associations between moral distress and the following demographic variables: age (*p*=0.02), gender (*p*=0.03), marital status (*p* < 0.001), educational level (*p*=0.02), and overall nursing experience (*p*=0.02). Specifically, moral distress was significantly higher among nurses aged 23–30, male nurses, single nurses, those holding a bachelor's degree, and nurses with 8–15 years of experience compared to their counterparts.

The mean score for moral disengagement among the nurses was 39 ± 10.9. The data showed a significant relationship between moral disengagement and several variables, including age (*p*=0.20), hospital type (*p*=0.40), overall nursing experience (*p*=0.10), and emergency department experience (*p*=0.10). In particular, nurses aged 23–30, those working in public hospitals, and those with 1–3 years of experience in both nursing and the emergency department exhibited significantly higher levels of moral disengagement. Furthermore, Pearson's correlation analysis revealed a significant and direct relationship between organizational bullying and moral distress (*p*=0.0112, *r* = 0.1865) at the 95% confidence level. A similarly significant and direct association was found between organizational bullying and moral disengagement (*p*=0.0006, *r* = 0.2358). Additionally, there was a significant and direct correlation between moral distress and moral disengagement (*p* < 0.001, *r* = 0.6470) (Table 2).

**Figure 1:**
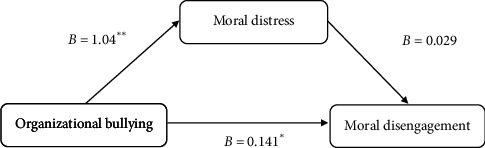
Mediation model illustrating the effect of moral distress on the relationship between organizational bullying and moral disengagement.

The mediation analysis of moral distress revealed that both the direct and indirect effects of organizational bullying on moral disengagement are significant. Specifically, the direct effect of organizational bullying on moral disengagement was found to be significant (*p* < 0.05, *B* = 0.141). Additionally, an indirect effect through moral distress was also identified (*p* < 0.05, *B* = 0.029; *p* < 0.001, *B* = 1.04), indicating that moral distress partially mediates this relationship (Figure 1).

## 4. Discussion

The present study was conducted in 2024 with the objective of examining the relationship between organizational bullying and both moral distress and moral disengagement among nurses working in the emergency departments of hospitals in Ilam Province. The mean score for experienced organizational bullying among the participants was 42 ± 14.5. This finding aligns with the results of Hidaya et al., in which 37.4% of the participating nurses reported experiencing organizational bullying [40]. Several studies conducted across various countries—including the United States, Canada, Turkey, China, the United Kingdom, and Australia—have reported varying prevalence rates of organizational bullying [41–46]. Furthermore, some research studies have indicated that certain participants consider bullying in the nursing environment an inevitable and inseparable aspect of the profession [47–49].

A multitude of factors can contribute to the high prevalence of organizational bullying. Particularly in Middle Eastern countries, the lack of a clear organizational structure, staffing shortages, ambiguous nursing duties, ineffective teamwork, and the lack of preventive and managerial policies have all contributed to the rise of this phenomenon [38–40]. Furthermore, Vessey et al. described bullying as a learned behavior that is intrinsically linked to the work environment [41].

One of the most significant factors influencing the reported prevalence of organizational bullying is how individuals interpret and define this phenomenon across different cultures. Another key reason for the discrepancies in reported prevalence rates is the variation in questionnaires and measurement methods. Differences in measurement criteria, the scope of definitions, and research instruments can serve as key factors in the divergent results observed across studies. Additionally, numerous studies have shown that organizational bullying is more prevalent in hospital emergency departments compared to other departments, with differences in professional status and job hierarchies in emergency settings also playing an influential role.

Another explanation for the variation in reported rates of organizational bullying across different countries is related to managerial policies and organizational support systems. In Iran, for instance, the absence of transparent policies to combat bullying and the lack of effective reporting systems may have allowed bullying behaviors to persist without legal repercussions, eventually becoming an accepted norm in nursing environments. Conversely, challenging working relationships between nurses and other healthcare professionals (such as physicians and hospital administrators) can further elevate tension and foster the emergence of domineering and bullying behaviors.

It is important to note that in the present study, although the mean organizational bullying score was reported, the quantitative nature of the questionnaire and the absence of clearly defined categories for bullying levels hindered the precise determination of the prevalence of this phenomenon. In contrast, other studies have reported the prevalence and level of bullying owing to differences in measurement instruments. Therefore, an analytical comparison was made between the findings of this research and those of various other studies. The discrepancies observed among different studies highlight the need for more comprehensive and precise investigations.

The findings of the present study also revealed that the mean moral distress score among nurses was 33 ± 41.4, and when considering both components, the nurses were categorized as experiencing low levels of moral distress. Several studies have reported similarly low levels of moral distress, which align with the results of the current study [50–54]. However, some research studies have produced results that differ from those found in this study [55–57], highlighting an inconsistency. This divergence in study outcomes can be attributed to various factors, including differences in work environments, job conditions, unequal positions, cultural variations, individual characteristics, and the internal experience of moral distress.

Overall, the results of various studies indicate that the prevalence and severity of moral distress among nurses working in different departments and countries vary considerably. The occurrence of this phenomenon fluctuates across different hospital units and nations. These discrepancies may stem from factors such as the type of questionnaires used, study populations, countries where research is conducted, the nature of the nursing profession, work experience, and other related factors.

Moreover, given the specific conditions of Ilam Province, which has high rates of poverty and suicide, socioecological factors play a very important role in shaping moral distress. These factors include the social structure of the region, the extent of people's access to resources and healthcare facilities, available social supports, and the difficult economic and cultural conditions affecting daily life. Additionally, since Ilam was one of the regions severely affected by the 8-year Iran–Iraq war, many families and communities have been directly or indirectly impacted by this conflict. The psychological and social consequences of this war are still felt within the community. This historical experience has imposed additional psychological and social pressures on individuals, including nurses who are on the frontline of healthcare.

Together, these socioecological factors may contribute to increased moral distress among nurses, as resource limitations, social and psychological challenges in the community, and insufficient support structures influence their work environment and daily lives, potentially reducing the quality of nursing care and their mental health. Therefore, moral distress directly affects the quality of nursing care, and its intensity may vary across different work environments and social conditions.

The findings revealed that the mean moral disengagement score among nurses was 39 ± 10.9. It appears that, in this study, nurses generally did not experience significant levels of moral disengagement. This may suggest improvements in ethical education and organizational support within hospitals, potentially reflecting the presence of educational programs, counseling services, or even ethical support systems in these institutions.

The conditions in emergency departments—especially in hospitals located in different regions—might lead to variations in moral disengagement. However, in this study, the mean score indicates that the working conditions for nurses in this particular region have not substantially contributed to this phenomenon. The results suggest that nurses working in the emergency departments of hospitals in Ilam Province in 2024 are in relatively good standing regarding moral disengagement. Nevertheless, further investigation is warranted, as even low levels of moral disengagement could gradually affect the specific quality of care under certain circumstances.

The results demonstrated a significant relationship between organizational bullying and moral distress (*p*=0.0112, *r* = 0.1865). The study's findings indicated a statistically significant, albeit weak to moderate, association between organizational bullying and moral distress. While no study has directly compared these two variables, it can be argued that organizational bullying may act as an exacerbating factor for moral distress.

Bullying can transform the work environment for nurses into a hostile setting where ethical decision-making becomes increasingly challenging. These decisions may involve issues that conflict with their personal and professional values. In such circumstances, moral distress emerges as a response to these internal conflicts. In fact, while organizational bullying might be one of the factors influencing moral distress, it is likely that other factors also contribute. Further research is needed to identify and examine these additional contributing factors.

The findings indicated a positive and significant relationship between organizational bullying and moral disengagement. In the study by Kowalski et al., a positive association between bullying and moral disengagement was also observed [58]. Likewise, studies by Haddock and Jimerson, Killer and et al., and Wang et al. have confirmed the link between organizational bullying and moral disengagement. These studies clearly demonstrate that bullying behaviors not only affect the victims but also influence bystanders and perpetrators, ultimately affecting the level of moral disengagement in different individuals. Notably, perpetrators of bullying often leads to the highest levels of moral disengagement, as they engage in behaviors that conflict with ethical principles [59–61].

This suggests that the more frequently nurses experience organizational bullying, the higher their levels of moral disengagement tend to be. In this context, it can be argued that emergency departments, with their unique work environments characterized by high workloads, psychological stress, and rapid, sensitive interactions with patients, provide a conducive environment for the emergence of moral disengagement. In summary, the results of this study underscore that organizational bullying can have serious repercussions on nurses' moral disengagement, particularly in high-stress environments such as emergency departments.

Based on the findings of the present study, there exists a significant and direct relationship between moral distress and moral disengagement, meaning that as moral distress increases, so does moral disengagement. This may imply that moral distress not only influences nurses' professional decision-making but can also directly affect their ethical behaviors, leading to deviations from established ethical standards. When this distress persists and nurses are unable to cope effectively, moral disengagement may eventually occur.

## 5. Conclusion

According to the study's findings, the nurses under investigation had comparatively low levels of moral anguish, moral disengagement, and organizational bullying. This can be linked to the adoption of suitable management policies and practices in Ilam Province's healthcare facilities, which have successfully stopped organizational bullying from happening and its negative consequences on employees.

Interestingly, 57.1% of the participants were male, which is unusual in Iran's traditionally female-dominated nursing workforce. This gender anomaly may have important implications for the dynamics of the emergency departments studied. Gender differences can influence communication styles, coping mechanisms, and responses to workplace stress and bullying. Moreover, cultural factors and societal expectations regarding gender roles could shape how male nurses experience moral distress and disengagement differently from their female counterparts. The higher proportion of male nurses in these settings may reflect specific demands or preferences related to the high-stress, fast-paced environment of emergency care. Understanding the impact of gender composition on moral distress and organizational behavior in nursing units is essential for tailoring interventions and support systems.

The study's findings supported the research hypotheses. The first hypothesis—which emphasized the relationship between organizational bullying and moral distress—was significantly confirmed. This outcome indicates that in work environments marked by bullying behavior, employees are more likely to experience moral distress. Moral distress, which arises from the conflict between personal values and workplace behavior, can negatively affect the mental well-being of nurses and compromise the quality of care provided.

The second hypothesis, addressing the relationship between organizational bullying and moral disengagement, was also confirmed. These results suggest that continuous exposure to organizational bullying may erode the ethical standards of employees.

Furthermore, the third hypothesis concerning the link between moral distress and moral disengagement was supported. This finding demonstrates that the inability to reconcile personal values with unfavorable working conditions can lead to diminished ethical commitment, ultimately reducing the quality of service and increasing unethical behavior in the workplace.

The study also revealed that organizational bullying influences moral disengagement both directly and indirectly through the elevation of moral distress. In this regard, moral distress acts as a mediating mechanism that facilitates the connection between organizational bullying and moral disengagement.

Overall, these results underscore the critical importance of establishing supportive work environments, enforcing antibullying policies, and implementing intervention programs to alleviate moral distress among staff. Initiatives such as ethics training, the cultivation of an organizational culture based on respect, and the development of effective reporting systems could help mitigate the negative effects of organizational bullying and enhance the psychological and ethical well-being of nurses. Moreover, the findings highlight the need for further research to identify additional factors influencing these relationships and to propose practical solutions for future studies.

## 6. Study Limitations

Some nurses were reluctant to participate due to fears of being identified and having their information disclosed. To address these concerns, confidentiality and the anonymity of the questionnaires were strictly assured to encourage participation. The lack of full psychometric validation of the translated Moral Disengagement Scale within the Iranian context is a limitation of the present study and should be addressed in future research.

## Figures and Tables

**Table 1:** Demographic characteristics of the participating nurses.

		*N*	Percentage (%)
Age (years)	23–30	147	55.2
	31–40	84	31.7
	41–50	29	10.9
	51–60	6	2.2

Sex	Female	114	42.9
	Male	152	57.1

Marital status	Single	155	58.3
	Married	111	41.7

Education	Bachelor's	191	71.8
	Master's	74	27.8
	Doctorate	1	0.4

Shift status	Rotating	248	93.2
	Fixed	18	6.8

Nursing experience (years)	< 1	4	1.5
	1–3	84	31.6
	4–7	70	26.3
	8–15	83	31.2
	> 15	25	9.4

Emergency department experience (Years)	< 1	10	3.8
	1–3	145	54.5
	4–7	64	24.0
	8–15	41	15.4
	> 15	6	2.3

Hospital type	Public	226	85.0
	Private	40	15.0

**Table 2:** Relationship between organizational bullying, moral distress, and moral disengagement.

		Organizational bullying	Moral distress	Moral disengagement
Organizational bullying	Pearson correlation coefficient	1		
	Sig	—		

Moral distress	Pearson correlation coefficient	0.1865	1	
	Sig	0.0112	—	

Moral disengagement	Pearson correlation coefficient	0.2358	0.6470	1
	Sig	0.0006	< 0.001	—

## Data Availability

The datasets used during the current study are available from the corresponding author upon reasonable request.

## Nomenclature

WHO: World health Organization
